# Voriconazole-Based Salts Are Active against Multidrug-Resistant Human Pathogenic Yeasts

**DOI:** 10.3390/molecules24203635

**Published:** 2019-10-09

**Authors:** Emil Szepiński, Dorota Martynow, Piotr Szweda, Maria J. Milewska, Sławomir Milewski

**Affiliations:** 1 Department of Organic Chemistry, Gdańsk University of Technology, 11/12 G. Narutowicza Str., 80-233 Gdańsk, Poland; e.szepinski@gmail.com (E.S.); marmilew@pg.edu.pl (M.J.M.); 2 Department of Pharmaceutical Technology and Biochemistry, Gdańsk University of Technology, 11/12 G. Narutowicza Str., 80-233 Gdańsk, Poland; dorota.koperkiewicz@gmail.com (D.M.); piotr.szweda@pg.edu.pl (P.S.)

**Keywords:** voriconazole, *Candida albicans*, multidrug resistance

## Abstract

Voriconazole (VOR) hydrochloride is unequivocally converted into VOR lactates and valinates upon reaction with silver salts of organic acids. This study found that the anticandidal in vitro activity of these compounds was comparable or slightly better than that of VOR. The *Candida albicans* clinical isolate overexpressing *CaCDR1/CaCDR2* genes, highly resistant to VOR, was apparently more susceptible to VOR salts. On the other hand, the susceptibility of another *C. albicans* clinical isolate (demonstrating multidrug resistance due to the overexpression of *CaMDR1*) to VOR salts was comparable to that to VOR. Comparative studies on the influence of VOR and its salts on Rhodamine 6G efflux from susceptible and multidrug-resistant *C. albicans* cells revealed that VOR salts are poorer substrates for the CaCdr1p drug efflux pump than VOR.

## 1. Introduction

The emergence of human pathogenic fungi that are resistant to commonly used antifungal drugs is a great challenge for antimicrobial chemotherapy [1]. Another problem is the limited repertoire of effective low-toxic antifungals for humans [2]. One such drug, voriconazole (VOR), is a second generation triazole antifungal agent, demonstrating high in vitro and in vivo activity against human pathogenic yeasts and molds. It is used in clinics to treat invasive aspergillosis and candidiasis, and fungal infections caused by *Scedosporium* and *Fusarium* species in immunocompromised patients [3]. It is a relatively safe drug and has a small number of side-effects with oral or intravenous administration [4]. Unfortunately, fungal resistance to VOR is not rare. The molecular basis of this resistance is most often a point mutation (especially in *Aspergillus* spp. Molds) in the *CYP51A* gene encoding lanosterol demethylase, which is an intracellular target for VOR [5]. However, resistance of *Candida* spp. yeast to VOR may also be due to the multidrug (MDR) type, owing to the overexpression of genes encoding drug efflux proteins belonging to the ABC or MFS subfamily. Evidence for participation of ABC transporters, CaCdr1p and CaCdr2p, in VOR resistance in *Candida albicans* were previously presented [6].

Earlier reports indicated that the conversion of azole and triazole antifungals—ketoconazole, fluconazole, tebuconazole and propiconazole—into respective ionic forms upon protonation of the nitrogen atoms in the azole or triazole ring and combination with the appropriate anion may result in improvement of the antifungal properties of the obtained salts, in comparison with the mother compounds [7,8,9]. Some of these salts exhibit physicochemical properties that are attributed to ionic liquids, especially the liquid state of matter at room temperature [7,8]. It is worth mentioning, therefore, that some types of ionic liquids are considered promising candidates for novel antifungals [10]. On the other hand, an improvement in antibacterial properties for water-soluble silver(I) complexes of metronidazole and selected counter-ions have been observed [11], and ampicillin-based ionic liquids have demonstrated paradoxical antitumor activity [12].

In this work, we present the results of our studies on preparation of VOR salts and their antifungal properties, including activity against human pathogenic yeasts exhibiting the MDR phenotype.

## 2. Results and Discussion

### 2.1. Chemistry

**Scheme 1:**
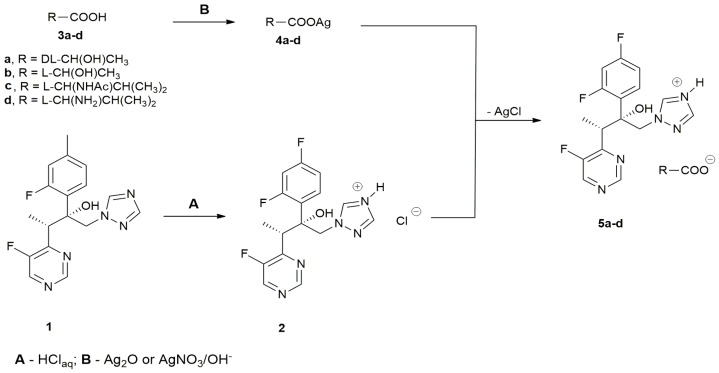
Synthesis of VOR salts.

An initial assumption of this study was preparation of salts consisting of the protonated VOR as a cation and a non-toxic organic acid as an anion. The VOR salts were synthesized by the original procedure shown in Scheme 1. 

VOR hydrochloride **2** was prepared directly from VOR (route A). VOR-based salts with organic acids **5a**–**d** were obtained by anion exchange upon reaction of **2** with silver salts of lactic acid, l-valine, and *N*-acetyl-l-valine; prepared by a reaction of an organic acid with Ag_2_O, added directly to the reaction mixture or generated in situ from AgNO_3_ and LiOH (route B). The yields of the final anion exchange reactions were stoichiometric and an identity of the VOR salts obtained was confirmed by ^1^NMR spectroscopy and mass spectrometry. 

The physicochemical properties of the VOR salts obtained are shown in Table 1. The salts with organic acids **5a**–**d** were white solids at room temperature, with melting points in the 119–203 °C range. Since the melting points of these salts were higher than 100 °C, none of them can be considered a protic ionic liquid. Only for [VOR][Cl] **2** were we able to determine the glass transition temperature; decomposition of all salts occurred well above 250 °C. These properties are mostly different from those of salts of products other than VOR azole antifungals, as reported by other authors. Fluconazole ascorbates or ketoconazole citrates were claimed to be red-colored liquids [7,8]. However, these salts were prepared by solvent evaporation from methanolic solutions of antifungal azole/organic acid mixtures at a 1:1, 1:2.5, or 1:5 molar ratio; therefore, the final composition of the preparations thus obtained were complex. Some of the salts of tebuconazole and propiconazole with different inorganic and organic anions appeared as ionic liquids, but most of them were solids [9]. Our methodology of VOR salt preparation ensures that the final products are unequivocally ionic compounds at a cation:anion 1:1 molar ratio. 

### 2.2. Antifungal Activity of VOR Salts

The antifungal in vitro activity of VOR and its salts against five reference strains of human pathogenic yeasts of the *Candida* genus was determined by the serial dilution method in microtiter plates under conditions recommended by CLSI [13], except for the spectrophotometric (not visual) readout. This modification was used to avoid problems resulting from the observed “trailing” effect of VOR and its salts, i.e., residual yeast growth at high compound concentrations. The minimal inhibitory concentrations (MIC) values are shown in Table 2. 

Activity of all VOR salts **2** and **5a**–**d** against *C. albicans, C. krusei, C. tropicalis*, and *C. lusitaniae* was identical or only slightly different (mostly higher) from that of VOR, whereas *C. glabrata* cells were markedly more susceptible to VOR salts than to VOR itself. This is consistent with data provided by Pernak et al., who found that the antifungal activity of salts of azole antifungals was similar to that of the mother compounds [9]. On the other hand, preparations obtained from fluconazole and ascorbic acid demonstrated same or lower activity against *Candida albicans* [7].

The growth inhibitory activity of VOR and its salts was also determined against clinical *Candida albicans* isolates Gu4, Gu5, B3, and B4. The Gu5 and B4 isolates are resistant to fluconazole (FLU), due to the FLU-induced overexpression of genes encoding drug efflux pumps, i.e., *CDR1/CDR2* in Gu5 and *MDR1* in B4 [14]. Their FLU-susceptible counterparts, Gu4 and B3, exhibit a basal expression of these resistance genes. The data shown in Table 3 clearly indicate that the MDR strains Gu5 and B4 were less susceptible to VOR than their Gu4 or B3 counterparts, and this difference was especially pronounced for the Gu4:Gu5 pair. Susceptibility of the B3 and B4 cells to VOR salts **2** and **5a**–**d** was nearly identical as to VOR, whereas the MIC values of VOR salts against the Gu5 strain were 8–16 times lower than that of VOR, while the MIC values against the Gu4 strain were only 2–4 times lower. These results show that the *C. albicans* MDR, due to overexpression of the *CDR1/CDR2* genes, can be more effectively overcome by VOR salts than by VOR itself. 

### 2.3. VOR Salts Are Poorly Effluxed by ABC Drug Transporters of C. albicans

**Figure 1:**
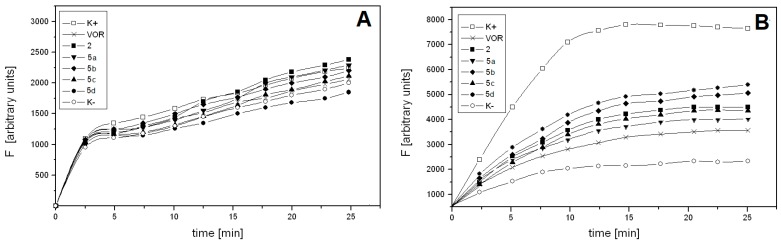
Time course of Rhodamine 6G efflux from *C. albicans* Gu4 (**A**) or Gu5 (**B**) cells. Glucose-starved cells were preloaded with Rhodamine 6G (10 μM) and 100 μM VOR or one of the VOR salts, if indicated. The probe and drug efflux was induced by glucose addition. Fluorescence intensity (F) was measured in supernatants collected at time intervals after cell harvesting.

To ascertain the reason for the relatively high activity of VOR salts against *CDR1/CDR2*-overexpressing *C. albicans* cells, the effect of VOR or VOR salt presence on the efflux of the fluorescent probe from Gu4 and Gu5 cells was measured. The probe used in this experiment was Rhodamine 6G (R6G), known as a good, selective substrate of the ABC-type drug efflux pump [15]. The cells, de-energized due to pre-incubation in the presence of 2-deoxy-d-glucose, were then loaded for 90 min with R6G and with VOR or a VOR salt at 10-fold molar excess to the fluorescent probe. The efflux was triggered by glucose addition, and the R6G pumped out of the cells was quantified spectrophotometrically. The results of this experiment are shown in Figure 1. 

The time-dependent R6G efflux from Gu5 cells, triggered by glucose addition, demonstrated saturation kinetics and reached maximum at ~15 min. The respective efflux from Gu4 cells slowly increased and its rate was 4–5 times lower. The difference in probe efflux rates from glucose-triggered (K+) and non-triggered (K−) Gu4 cells was negligible and the presence of VOR or any of its salts had very little, if any effect on this efflux (Figure 1A). On the contrary, the glucose-triggered (K+) Gu5 cells extruded about five times more of the probe than the non-triggered (K−) ones. VOR effectively competed with R6G for the glucose-triggered efflux from Gu5 cells, as the RG6 efflux rate in the presence of VOR was only slightly higher than that from the K− cells (Figure 1B). All the VOR salts were less effective than VOR as RG6 competitors, what suggests that VOR salts are poorer than VOR substrates for Cdr1p/Cdr2p drug efflux pumps. This result offers a reasonable explanation for the observed high activity of VOR salts against *CDR1/CDR2* overexpressing Gu5 cells (Table 3).

The molecular basis for the difference between VOR and its salts in their affinity to Cdr1p and Cdr2p is not clear. The ABC-type drug transporters of *C. albicans* are known for their broad substrate spectrum. Many structurally unrelated (usually polycyclic aromatic compounds) containing atom-centered fragments were identified as good substrates of Cdr1p and Cdr2p [16]. Most of them are not charged, but a few examples of ionic compounds, including Rhodamine 6G and berberine hydrochlorides, are known. 

We were unable to compare the affinity of VOR and its salts to Mdr1p, overexpressed in B4 strain cells, because fluorescent probes demonstrating selective affinity to this drug efflux protein are not known. Nevertheless, the little, if any, difference in susceptibility of B3 and B4 cells to VOR and its salts (Table 3) suggests that these affinities must be very similar. 

## 3. Materials and Methods

### 3.1. Preparation of Voriconazole-Based Salts

All materials were purchased from commercial sources and used without further purification. ^1^H NMR spectra were recorded on a Varian Unity 500 plus (500 MHz) and Bruker AV400 (400 MHz) spectrometers in CD_3_OD or D_2_O with TMS as standard. The mass spectra analyses were carried out using the Aqilent Technologies 6540 UHD Accurate–Mass Q-TOF LC/MS spectrometer (Santa Clara, CA, USA).

**Voriconazole hydrochloride [VOR][Cl] (2):** Voriconazole **1** (1 mmol, 349 mg) was suspended in 3 mL of water in round-bottomed flask equipped with a magnetic stirrer. Concentrated HCl (2 mmol) was added dropwise for 10 min and stirring was continued for another 1 h at room temperature. The solvent was removed under reduced pressure and the product was dried under the same conditions. Yield 100%. 

HRMS-ESI: ES⁺ *m*/*z*: 350.1245; calc. for C_16_H_15_F_3_N_5_O^+^ 350.1223

***N*-Acetyl-l-Valine (3b)** was synthesized according to the procedure described by Simons et al. [17].

**Silver dl-lactate (4a) and silver l-lactate (4b):** The reaction was run under dark conditions. dl-lactic acid or l-lactic acid **3a** (4.4 mmol, 396 mg) was dissolved in 3 mL of methanol in **a** round-bottomed flask equipped with a magnetic stirrer and reflux. Ag_2_O (2.2 mmol, 510 mg) and a drop of 3% H_2_O_2_ were added and the reaction was run overnight at 60 °C, with constant stirring. Afterwards, the mixture was chilled; the resulting grey precipitate was washed with acetonitrile and dried under reduced pressure at 50 °C. The yield was 50%, mp 120–121 °C. 

HRMS-ESI: ES^−^ *m*/*z*: 89.0241; calc. for C_3_H_5_O_3_^−^ 89.0244

**Silver *N*-acetyl-l-valinate (4c):** The reaction was run under dark conditions. Ag_2_O (0.76 mmol, 176 mg) was suspended in 4 mL of acetonitrile in **a** round-bottomed flask equipped with a magnetic stirrer. *N*-acetyl-l-valine **3b** (1.5 mmol, 239 mg) was added and the reaction was run overnight at room temperature, with constant stirring. Afterwards, the precipitate was filtered, washed with acetonitrile and diethyl ether, and dried under reduced pressure at 50 °C. The yield was 30%. 

HRMS-ESI: ES^−^ *m*/*z*: 158.0864; calc. for C_7_H_12_NO_3_^−^ 158.0823

**Silver l-valinate (4d):** The reaction was run under dark conditions. AgNO_3_ (1 mmol, 239 mg) was dissolved in 3 mL of MeOH/H_2_O 8:2 *v/v* mixture in a round-bottomed flask equipped with a magnetic stirrer. l-Valine (1 mmol, 117 mg) was added, followed by LiOH (1 mmol, 24 mg). The reaction was run overnight at room temperature, with constant stirring. Afterwards, the precipitate was filtered, washed with acetonitrile, and dried under reduced pressure at 50 °C. The yield was 30%. 

HRMS-ESI: ES^−^ *m*/*z*: 116.0692; calc. for C_5_H_10_NO_2_^−^ 116.0717

**Voriconazole dl-lactate [VOR][dl-Lac] (5a)** and **voriconazole l-lactate [VOR][l-Lac] (5b):** The reaction was run under dark conditions. Voriconazole hydrochloride **2** (1 mmol, 386 mg) was dissolved in 3 mL of MeOH/H_2_O 1:1 *v/v* mixture in a round-bottomed flask equipped with a magnetic stirrer. Silver dl-lactate **4a** or silver l-lactate **4b** (1 mmol, 197 mg) was added and the reaction was run overnight at room temperature, with constant stirring. Afterwards, the resulting AgCl precipitate was filtered and washed with methanol and water. The washes were combined with the filtrate, the solvents were evaporated, and the resulting product was dried under reduced pressure at 50 °C. The yield was 100%. 

HRMS-ESI: ES^+^ *m*/*z*: 350.1248; calc. for C_16_H_15_F_3_N_5_O^+^ 350.1223

     ES^−^ *m*/*z*: 89.0281; calc. for C_3_H_5_O_3_^−^ 89.0244

^1^H NMR (500 MHz, CD_3_OD) δ 9.01 (d, *J* = 2.5 Hz, 1H), 8.77 (d, *J* = 1.8 Hz, 1H), 8.32 (s, 1H), 7.62 (m, 1H), 7.49 (m, 1H), 6.98 (m, 1H), 6.86 (t, *J* = 8.4 Hz, 1H), 4.87 (d, *J* = 14.3 Hz, 1H), 4.38 (d, *J* = 14.2 Hz, 1H), 4.26 (s, 1H), 4.17 (q, *J* = 7.1 Hz, 1H), 1.42 (m, 3H), 1.15 (t, *J* = 7.0 Hz, 3H).

**Voriconazole *N*-acetyl-l-valinate [VOR][Ac-l-Val](5c):** The reaction was run analogously as for the preparation of **5a** and **5b**. From 1 mmol (386 mg) of voriconazole hydrochloride **2** and 1 mmol (266 mg) of silver *N*-acetyl-l-valinate **4c**, a stoichiometric amount of the **5c** product was obtained. 

HRMS-ESI: ES^+^ *m*/*z*: 350.1212; calc. for C_16_H_15_F_3_N_5_O^+^ 350.1223

     ES^−^ *m*/*z*: 158.0564; calc. for C_7_H_12_NO_3_^−^ 158.0823

^1^H NMR (500 MHz, CD_3_OD) δ 9.81 (s, 1H), 9.10 (d, *J* = 2.4 Hz, 1H), 8.83 (s, 1H), 8.57 (m, 1H), 7.44 (m, 1H), 7.06 (m, 1H), 6.90 (t, 1H), 5.09 (d, *J* = 14.1 Hz, 1H), 4.57 (d, *J* = 14.1 Hz, 1H), 4.32 (d, *J* = 6.0 Hz, 1H), 4.20 (q, 1H), 2.17 (m, 1H), 2.03 (s, 3H), 1.19 (d, *J* = 7.1 Hz, 3H), 0.97 (d, *J* = 2.9Hz, 6H).

**Voriconazole l-valinate [VOR][l-Val] (5d):** The reaction was run analogously as for the preparation of **5a** and **5b**. From 1 mmol (386 mg) of voriconazole hydrochloride **2** and 1 mmol (224 mg) of silver l-valinate **4d**, a stoichiometric amount of the **5d** product was obtained. 

HRMS-ESI: ES^+^ *m*/*z*: 350.1251; calc. for C_16_H_15_F_3_N_5_O^+^ 350.1223

     ES^−^ *m*/*z*: 116.0722; calc. for C_5_H_10_NO_2_^−^ 116.0717

^1^H NMR (500 MHz, CD_3_OD) δ 9.01 (d, *J* = 2.1 Hz, 1H), 8.77 (d, *J* = 2.0 Hz, 1H), 8.28 (s, 1H), 7.68 (s, 1H), 7.39 (m, 1H), 7.04 (m, 1H), 6.89 (m, 1H), 4.92 (d, *J* = 14.2 Hz, 1H), 4.37 (d, *J* = 14.6 Hz, 1H), 4.17 (q, *J* = 7.1 Hz, 1H), 3.56 (d, *J* = 4.4 Hz, 1H), 2.33 (m, 1H), 1.15 (d, *J* = 6.8 Hz, 3H), 1.03 (d, *J* = 6.8 Hz, 6H).

### 3.2. Determination of Physicochemical Properties 

Melting point values were obtained by visual observation via hot-plate apparatus. Glass transition temperatures were determined by DSC, with a Mettler Toledo Stare DSC1 (Leicester, UK) unit, under nitrogen, at a heating rate of 10 °C·min^−1^.

### 3.3. Microorganisms and Growth Conditions 

The reference human pathogenic yeasts used in this study were: *Candida glabrata* DSM 11226, *Candida albicans* SC 5314, *Candida lusitaniae* DSM 70102, *Candida krusei* DSM 6128, and *Candida tropicalis* KKP 334. *C. albicans* B3, B4, Gu4, and Gu5 clinical isolates were gifts from Joachim Morschhäuser, Würzburg, Germany. The strains were grown in YPD medium (2% glucose, 1% Yeast Extract and 1% Bacto Peptone) at 30 °C and stored on YPD agar plates containing 2% agar.

### 3.4. Determination of Antifungal In Vitro Activity

Minimal inhibitory concentration (MIC) values were determined by the serial two-fold dilution method in RPMI-1640 medium, using the 96-well microtiter plates, as per CLSI recommendations [13], except for the end-point readout that was done by spectrophotometric determination of turbidity, measured with a microplate reader (Victor^3^; Perkin Elmer) at 531 nm. MIC was defined as the lowest compound concentration that resulted in at least 80% decrease in turbidity, relative to that of the growth control.

### 3.5. Quantification of Rhodamine 6G Efflux from C. albicans Cells

The Rhodamine 6G (R6G) efflux from Gu4 or Gu5 cells was determined by a modified version of the previously described method [9,15,18]. Late exponential *C. albicans* cells grown in YPD medium were centrifuged (3000× *g*, 5 min, r.t.) and washed 3× with sterile water. The cells were then re-suspended in 50 mM potassium phosphate buffer, pH 6.0, containing 5 mM 2-deoxy-d-glucose, to OD_660_ ≈ 0.4. Cell suspensions were incubated for 60 min at 30 °C with shaking (50 rpm). The cells were then loaded with R6G (10 μM). One of the examined compounds was added (100 μM), when appropriate, and incubation was continued for 90 min. The cells were then washed 2× with phosphate buffer, pH 6.0, suspended in a fresh buffer to OD_660_ ≈ 0.2 and the resulting suspension was incubated at 30 °C for 5 min. d-Glucose was added (40 μM) and incubation was continued. 1-mL aliquots were collected at 2.5-min time intervals and centrifuged; 0.1 mL samples of the supernatants were transferred to the wells of a microtitrer plate. The fluorescence of the samples was measured with a Perkin-Elmer LS55 spectrofluorimeter (Perkin Elmer Inc., Wellesley, MA, USA), equipped with a microplate reader (excitation wavelength 529 nm and emission wavelength 553 nm). The negative control was a sample of the given cell suspension, treated as above, except for the glucose addition.

## 4. Conclusions

VOR lactates and valinates, prepared from VOR hydrochloride upon reactions with silver(I) lactate or silver(I) valinate, are white solids with relatively high melting points, well above 100 °C. Their antifungal in vitro activity against several species of human pathogenic yeasts from the *Candida* genus is same or slightly higher (against *C. glabrata*) than that of the mother antifungal. A notable difference is the much higher activity of the compounds than that of VOR activity of VOR salts against clinical *C. albicans* isolates. It demonstrates an MDR phenotype due to the overexpression of *CDR1/CDR2* genes, resulting in highly increased efficiency of the Cdr1p/Cdr2p drug efflux protein. The augmented susceptibility of the VOR-resistant *C. albicans* cells overexpressing *CDR1/CDR2* to VOR salts is a consequence of lower affinity, as compared to VOR of VOR salts to Cdr1p/Cdr2p. Therefore, the VOR salts described in this work seem to be promising candidates for antifungal drugs in the treatment of disseminated candidiasis caused by a particular type of multidrug-resistant *C. albicans*.

## Figures and Tables

**Table 1:** Physicochemical properties of VOR salts.

Comp.	T_g_ [°C]	mp [°C]	T_onset_ [°C]
[VOR][Cl] **2**	38	-	>250
[VOR][dl-Lac] **5a**	-	203	>250
[VOR][l-Lac] **5b**	-	119	>250
[VOR][Ac-l-Val] **5c**	-	130	>250
[VOR][l-Val] **5d**	-	176	>250

T_g_—glass transition temperature; mp—melting point; T_onset_—decomposition temperature.

**Table 2:** Fungistatic activity of VOR and its salts against selected *Candida* reference strains. MICs were determined by the microtiter serial twofold dilution method in RPMI-1640 medium.

Comp.	MIC (μg mL^−1^)				
	*C. albicans*	*C. glabrata*	*C. krusei*	*C. tropicalis*	*C. lusitaniae*
VOR **1**	1.0	4.0	2.0	0.25	0.062
[VOR][Cl] **2**	2.0	0.5	1.0	0.25	0.062
[VOR][dl-Lac] **5a**	1.0	0.5	1.0	0.125	0.031
[VOR][l-Lac] **5b**	0.5	0.5	1.0	0.25	0.031
[VOR][Ac-l-Val] **5c**	0.5	0.5	0.5	0.125	0.062
[VOR][l-Val] **5d**	0.5	0.5	1.0	0.125	0.062

**Table 3:** Comparison of antifungal in vitro activity of VOR and its salts against drug-susceptible (Gu4 and B3) and multidrug-resistant (Gu5 and B4) *Candida albicans* clinical strains. MICs were determined by the microtiter serial twofold dilution method in RPMI-1640 medium.

Comp.	MIC (μg·mL^−1^)			
	Gu4	Gu5	B3	B4
VOR **1**	0.125	4.0	1.0	4.0
[VOR][Cl] **2**	0.031	0.5	1.0	4.0
[VOR][dl-Lac] **5a**	0.062	0.5	0.5	2.0
[VOR][l-Lac] **5b**	0.031	0.25	0.5	2.0
[VOR][Ac-l-Val] **5c**	0.062	0.5	1.0	4.0
[VOR][l-Val] **5d**	0.031	0.25	0.5	2.0
